# Successful clozapine rechallenge following recurrent clozapine-associated pancreatitis: a case report

**DOI:** 10.1186/s40360-020-00413-6

**Published:** 2020-05-20

**Authors:** Victoria Rodriguez, Kieran Hanley, Ana Julia Arias, Diego Quattrone, Joseph Kuforiji, Eromona Whiskey, Sukhi S. Shergill

**Affiliations:** 1 Department of Psychosis Studies, Institute of Psychiatry, Psychology and Neuroscience, King’s College London, Denmark hill, London, SE5 9RS UK; 2 South London and Maudsley NHS Foundation Trust, London, UK; 3 Hospital de Salud Mental “El Sauce”, Mendoza, Argentina; 4 Clinical Research Fellow, Social Genetic & Developmental Psychiatry, King’s College London, and National Psychosis Unit, Bethlem Royal Hospital, London, UK; 5 National Psychosis Unit, Bethlem Royal Hospital, London, UK; 6 National Psychosis Service, and Pharmacy Department, South London and Maudsley NHS Foundation Trust, London, UK

**Keywords:** Clozapine, Schizophrenia, Side effects, Re-challenge, Acute pancreatitis

## Abstract

**Background:**

Acute pancreatitis is a rare but recognised complication of clozapine leading to termination of treatment.

**Case presentation:**

We present the case of a 39-year-old man with treatment-resistant schizoaffective disorder and a history of recurrent acute pancreatitis attributed to clozapine. After 15 years of unremitting symptoms with disruptive and aggressive behaviour, he was admitted for a clozapine rechallenge. Despite experiencing two further episodes of acute pancreatitis during clozapine treatment that led to its temporary withdrawal, clozapine was successfully re-established under gastroenterology consultation with close monitoring which resulted in progressively marked improvement of his mental state.

**Conclusions:**

This case demonstrates that patients who develop pancreatitis during clozapine treatment may be cautiously rechallenged with specialist gastroenterology support.

## Background

Clozapine is an second-generation antipsychotic recommended for treatment-resistant psychosis, which is defined by the lack of response to at least two different antipsychotics at therapeutic doses and for sufficient duration [1, 2]. The superior efficacy of clozapine in refractory schizophrenia is without doubt [3]. Nonetheless, its use entails the risk of some serious medical complications such as agranulocytosis, myocarditis, cardiomyopathy, gastrointestinal hypomotility leading to bowel infarction, renal insufficiency and acute pancreatitis among others. In this brief report, we describe the successful reintroduction after treatment cessation attributed to clozapine-induced pancreatitis.

Over 100 drugs have been implicated as causing acute pancreatitis [4], with around 0.1–2% of acute pancreatitis being labelled as drug-induced [5, 6]. Acute pancreatitis has been reported with virtually every antipsychotic including clozapine [7–10]. With other antipsychotics, this is of less importance as another agent can be substituted, but with clozapine, treatment cessation may be life changing and carries substantially greater significance.

Pancreatitis is a potentially life-threatening drug adverse effect resulting in treatment cessation in almost all cases. In an recent review of 41 cases, rechallenge was described in only 7% of cases [11]. Indeed, rechallenge after clozapine-induced pancreatitis is not recommended [12, 13]. Nonetheless, a recent case report showed a case of rechallenge after a clozapine-induce pancreatitis, although other factors, including polypharmacy may have been implicated [14]. Here we present a first case of successful complete clozapine rechallenge in a patient with a diagnosis of clozapine-induced pancreatitis.

## Case presentation

### Presentation

The patient is a 39-year-old Caucasian gentleman with a diagnosis of treatment resistant schizoaffective disorder. His symptoms were first observed at the age of 17, while he was regularly using cannabis and was first referred to psychiatric services at the age of 19 after presenting aggressive behaviour towards his parents. He presented with marked social withdrawal in the context of refusing to eat due to paranoid delusions about his food being poisoned. Various antipsychotic treatments failed to yield a satisfactory response.

He was eventually started on clozapine at the age of 25 years with the dose gradually titrated up to 400 mg daily together with lithium resulting in good clinical response. He was reported to be clinically stable until clozapine was discontinued due to non-compliance, leading to a rapid deterioration in his mental state. The duration of treatment on clozapine during this episode is unclear. A second clozapine trial was initiated at the age of 34 during an inpatient admission, while he was also taking lithium at therapeutic levels. After 2 weeks, he presented as lethargic, complaining of feeling physically unwell, experiencing shoulder and back pain, with repeated hiccups and flatulence and on physical examination a slight abdominal tenderness was observed. He was referred to the accident and emergency unit (A&E) with a temperature of 37.6 °C, WBC 21 × 10^9^/l and amylase of 900 U/L (ref 28–100 U/L), where he was diagnosed with acute pancreatitis attributed to clozapine, leading to clozapine discontinuation.

In 2015, aged 36 years, a third clozapine trial was commenced with good clinical response. At the time, he was being cross-titrated from olanzapine. Treatment with sodium valproate for mood stabilisation was continued. However, after 4 weeks he was admitted to hospital with another episode of pancreatitis. The CT scan described “interval accumulation of the head of pancreas pseudocyst causing duodenal obstruction and significant surrounding inflammation change”, requiring cholecystectomy, transduodenal trucut biopsies of the head of the pancreas and gastrojejunostomy. Due to the lack of evidence of presence of gallstones, hypertriglyceridemia or regular use of alcohol, and considering the previous episode, the patient was given a diagnosis of clozapine-induced pancreatitis and clozapine was discontinued once again.

After two further years of poor clinical response to other antipsychotics, the patient was eventually referred and admitted to the National Psychosis Unit (NPU), a tertiary referral unit in the United Kingdom, specialised in the management of refractory schizophrenia. At this point, he was prescribed olanzapine 20 mg daily and valproate semi-sodium 1250 mg twice daily. As he was very reluctant about taking clozapine, other pharmacological approaches with some evidence as alternatives to clozapine were first trialled including high dose olanzapine [15] and lurasidone with vortioxetine augmentation [16, 17]. These approaches failed to yield satisfactory clinical improvement. At this point and when facing such complex clinical scenarios, Electroconvulsive Therapy (ECT) should be considered, but as the affective symptoms were not prominent, we decided to not proceed with it as the next therapeutic option. Consequently, as clozapine was the only treatment with previous favourable response, an opinion from the gastroenterology team was sought. They advised restarting clozapine cautiously with twice weekly monitoring of LFTs and amylase levels. The significant risk of a further episode of pancreatitis associated with taking clozapine was also discussed with the family, who consented to proceed with the re-trial.

In his sixth month of hospitalization, he was admitted to a general hospital with an episode of haematuria with penile and abdominal pain, in which it was also noted that his amylase level was elevated to 508 U/L, remaining high for about a month. At the time he was taking olanzapine (20 mg OD) and sodium valproate (500 mg BD). CT findings at the time suggested a combination of acute and previous episodes of pancreatitis, in what would be his third pancreatitis episode.

Soon after the discharge from acute medical hospital and return to the NPU and in the absence of any current pancreatic contra-indications, he was started on clozapine. This time, both olanzapine (20 mg OD) and valproate 500 mg BD were gradually withdrawn before clozapine initiation. His amylase levels remained elevated and continued to fluctuate (from 100 to 505 U/L), but he was asymptomatic with respect to abdominal symptoms. FBC testing during this time was also normal. Nonetheless, after 2 months he experienced a fourth episode of suspected pancreatitis. At a dose of 250 mg a day and clozapine levels of 0.14 mg/dL (1400 ng/mL), the amylase levels rose to 282 U/L, coupled with a rise in Alkaline phosphatase (210 U/L) and Gamma glutamyl transferase (GGT) (267 U/L), although he was asymptomatic otherwise.

Given the successful clinical response and after discussing with the family and the gastroenterology team, the decision to continue clozapine was made with more frequent monitoring of his physical observations and bloods. Two months into clozapine treatment, with amylase level at 416 U/L, he complained of some non-specific lower back pain. However, 3 days later, with clozapine level of 0.26 mg/dL (2600 ng/mL), amylase was normalising at 154 U/L, and GGT and ALP were also reducing. The gastroenterology outpatient clinic reported that imaging had shown a thick-walled pancreatic pseudocyst and recommended a switch to an alternative medication if the amylase levels rose again above 200 U/L or if he became increasingly symptomatic with abdominal pain.

During the following 2 months, there were no concerns regarding the patient’s physical health and he continued to take clozapine without any significant side effects. His mental state continued to improve on clozapine and there were no incidents of verbal or physical aggression that characterised his illness prior to starting clozapine.

**Fig. 1:**
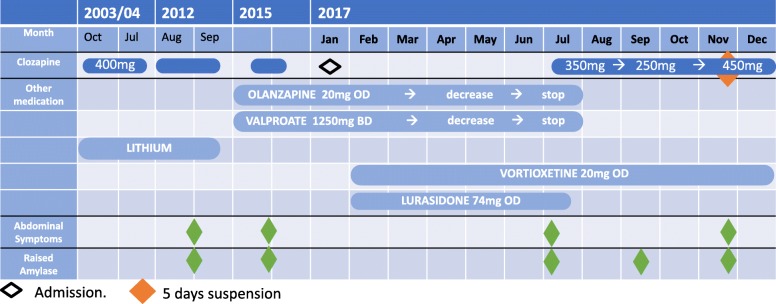
Gantt Chart presenting timeline of events. OD: once a day BD: twice a day.

After 4 months of clozapine treatment, the patient was admitted to the local hospital due to a new episode of haematuria, dysuria and penile and abdominal pain. At this time, blood testing showed two concerns: firstly low white cell (2.88 × 10^9^/l) and neutrophils (1.83 × 10^9^/l) count; and secondly a suspected fifth episode of acute pancreatitis with high amylase levels (up to 767 U/L). During the admission, CT imaging of his pancreas showed again an acute-on-chronic inflammation. Clozapine treatment was withheld for 5 days, due to the neutropenia according to clozapine monitoring guidelines, however clozapine was restarted as WBC and neutrophil resolved with the gastroenterology team support and agreement of the family. Amylase levels normalised regardless of clozapine being restarted, so it was felt that it would be safe for him to continue to take clozapine given the positive effects on his psychotic symptoms. A summary of changes of treatment alongside with abdominal symptoms and amylase levels is presented in Fig. 1.

### Outcome and follow-up

The patient was eventually discharged from the psychiatric ward 1 year after his admission in the NPU to a local psychiatric rehabilitation ward. He was discharged on a clozapine dose of 450 mg daily and serum clozapine levels of 0.18 mg/dL (1800 ng/mL). Except for the 5 days in the local general hospital, he had been taking clozapine consistently for 7 months with considerable improvement in terms of his clinical presentation and functionality. No hostility and aggressive behaviour were observed in the last period of his admission and his relationship with his family was reported to be markedly improved. The advice on discharge from the inpatient unit was to continue clozapine if the patient developed a new episode of acute pancreatitis and to treat the episode symptomatically maintaining the clozapine treatment if possible.

## Discussion and conclusion

We present a case of successful rechallenge in a patient with resistant schizoaffective after several episodes of acute pancreatitis suspected to be induced by clozapine.

So far, at least ten cases have been published on clozapine-induce pancreatitis resulting in drug withdrawal [18–22]. As mentioned before, pancreatitis is classed as one of the clozapine-induced complications considered to be non-re-challengeable [12]. However, in cases where there is no a valid alternative, a thorough evaluation of the risk versus benefit assessment with involvement of medical specialists is essential.

Idiopathic acute pancreatitis accounts for 10–40% of patients with acute pancreatitis [23, 24], while drug induced acute pancreatitis accounts only for 0.1–2% of all cases of acute pancreatitis [5, 6]. Additionally, many patients with idiopathic pancreatitis or microlithiasis have recurrent attacks of acute pancreatitis. Literature suggests that a first episode of acute pancreatitis leads to recurrent pancreatitis in 17% of patients, and almost 8% of patients progress to chronic pancreatitis within 5 years. (40). Therefore, as suggested by Girard in 1987, stopping and restarting a drug may coincide with recurrence of pancreatitis and not arise through cause and effect [25].

Also, pancreatitis can be triggered by several types of medication and the risk is increased further by polypharmacy. Cases of acute pancreatitis induced by other atypical antipsychotic and by sodium valproate [26–28] have been reported. As Sodium valproate and olanzapine were both discontinued before reintroducing clozapine ruling out valproate as a factor in the fourth episode of pancreatitis. In addition, pancreatitis complicated by a pancreatic pseudocyst have also been described [29, 30], as observed in our case.

In summary, we report a case of a patient with treatment refractory illness who was successfully treated with clozapine, after previously having previously experiences two episodes of acute pancreatitis attributed to the use of clozapine. To the best of our knowledge, this is the first case report showing a successful clozapine re-challenge after recurrent episodes of pancreatitis during clozapine titration. Pancreatitis causes are broadly heterogeneous and may be wrongly attributed to clozapine. Clinicians should weigh risks and benefits of rechallenge given limited therapeutic options in cases of treatment resistant schizophrenia, requiring in that case a careful case-by-case consideration with a multidisciplinary approach under close supervision of gastroenterology team. While we warn about the no generalisation of conclusions from scattered cases into clinical practice, our case report encourages further research to establish the safety to rechallenge clozapine after pancreatitis.

### Limitations

Serum lipase offers a higher sensitivity than serum amylase in diagnosing acute pancreatitis. Nonetheless, our local laboratory did not routinely measure lipase levels and this should be noted as a limitation.

## Data Availability

Not applicable.

## Abbreviations

A&E: Accident and emergency
WBC: White blood cell
CT: Computed tomography
NPU: National Psychosis Unit
LFT: Liver function test
OD: Once a day
BD: Twice a day
ALP: Alkaline phosphatase
GGT: Gamma-glutamyl transferase
ZTAS: Zaponex Treatment Access System
FBC: Full blood count
